# Monoclinic nonlinear metasurfaces for resonant engineering of polarization states

**DOI:** 10.1515/nanoph-2025-0019

**Published:** 2025-04-23

**Authors:** Ivan Toftul, Dhruv Hariharan, Pavel Tonkaev, Fangxing Lai, Qinghai Song, Yuri Kivshar

**Affiliations:** Research School of Physics, Australian National University, Canberra, ACT 2601, Australia; Ministry of Industry and Information Technology, Key Lab of Micro-Nano Optoelectronic Information System, Guangdong Provincial Key Laboratory of Semiconductor Optoelectronic Materials and Intelligent Photonic Systems, Harbin Institute of Technology, Shenzhen 518055, People’s Republic of China

**Keywords:** chiral metasurface, third-harmonic generation, nonlinear resonant metaphotonics

## Abstract

Polarization is a fundamental property of light that can be engineered and controlled efficiently with optical metasurfaces. Here, we employ *chiral metasurfaces* with monoclinic lattice geometry and achiral meta-atoms for resonant engineering of polarization states of light. We demonstrate, both theoretically and experimentally, that a monoclinic metasurface can convert linearly polarized light into elliptically polarized light not only in the linear regime but also in the nonlinear regime with the resonant generation of the third-harmonic field. We reveal that the ellipticity of the fundamental and higher-harmonic fields depends critically on the angle of the input linear polarization, and the effective chiral response of a monoclinic lattice plays a significant role in the polarization conversion.

## 1 Introduction

Among many remarkable achievements associated with the name Federico Capasso, metasurfaces play an important role as efficient planar components of future photonic devices [1], [2], [3]. One of the fundamental functionalities of metasurfaces is the control of polarization of light, which is a versatile degree of freedom that can be manipulated or engineered for numerous applications [4], [5]. Conventional approaches to manipulating polarization often rely on bulk components such as wave plates. Although effective, these components are constrained by their size.

Metasurfaces, composed of subwavelength structures, that is, meta-atoms, are powerful tools for overcoming size and weight limitations, allowing ultra-thin and multifunctional optical devices [6], [7], [8]. The operational principle for using metasurfaces in the control of polarization relies on transforming an incident waveform into an ensemble of individual beams generated by meta-atoms with different polarization states that beat along the optical axis, thereby changing the resulting polarization at will. Many recent papers have been devoted to the study of linear polarization conversion with single metasurfaces (see, e.g. Refs. [9], [10], [11], [12], [13]), as well as multiple metasurfaces with stacking and twisted configurations [14], [15]. Polarization transformations with metasurfaces have been extensively explored by the Federico Capasso group [16], [17], [18]. In particular, the designer-specified polarization response was shown to be employed for computer-generated holograms whose far-fields implement parallel polarization analysis and customized waveplates [18]. Additionally, full-stokes polarization encoding in metasurfaces has been demonstrated in both the near [19], [20] and far-field regimes [21], [22].

*Chiral metasurfaces* are particularly suitable for polarization engineering because of their inherit ability to mix polarization states, e.g. to convert directly linearly polarized light into elliptically or circularly polarized states [23]. The use of resonant effects in metasurfaces has been demonstrated to enhance polarization conversion efficiencies and enable functionalities such as circularly polarized lasing and high-contrast polarization detection [24], [25], [26] as well as achieving huge imbalance in the third harmonic intensity depending on the helicity of the input field [27].

**Figure 1:**
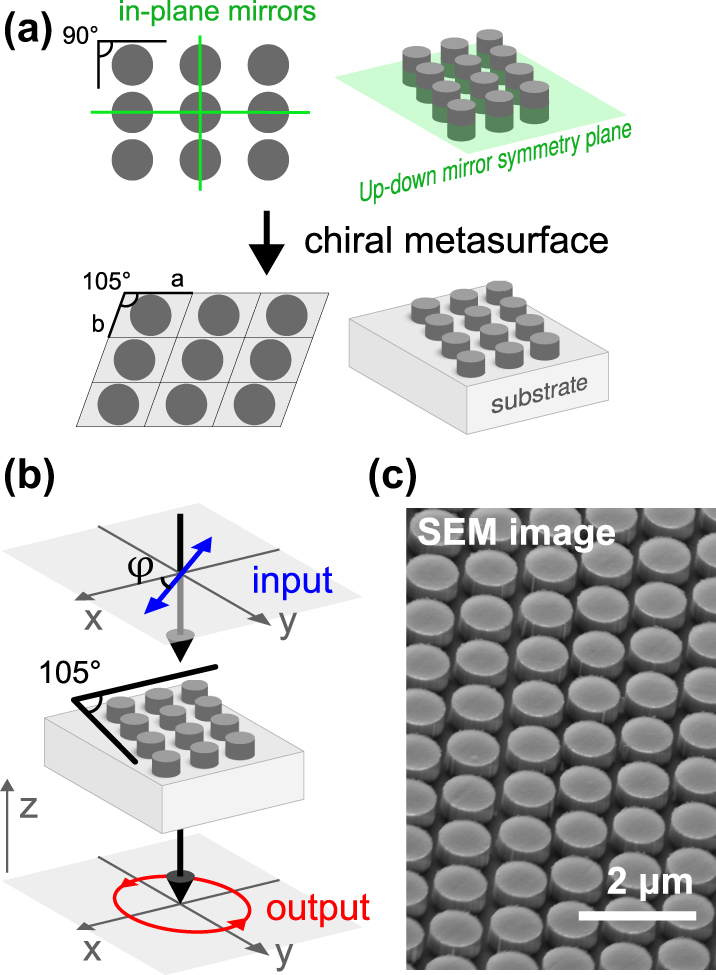
Concept of monoclinic chiral metasurfaces. (a) Design principle of the chiral metasurfaces utilizing monoclinic meta-atom lattices. (b) Schematic of a chiral metasurface which transforms incident linearly polarized plane wave into elliptically polarized light. (c) Scanning electron microscope (SEM) image of the fabricated metasurface.

In this work, we uncover the hidden potential of resonant chiral monoclinic metasurfaces, recently introduced and characterized in Ref. [28], for polarization conversion. While the previous study focused on circularly polarized input, this work explores the metasurfaces’ response from linearly polarized input, and expands this concept to the nonlinear polarization conversion for the generation of third-harmonic chiral fields (Figure 1). Using both computational and experimental approaches, we demonstrate their ability to convert linearly polarized light into elliptically polarized light in both the linear and nonlinear regimes. Specifically, we explore the third harmonic generation (THG) process and show that the ellipticity of the generated light is strongly dependent on the input polarization angle. By analyzing the role of chiral resonances, we underpin the underlying mechanisms that govern these effects, highlighting the versatility of monoclinic metasurfaces as compact polarization engineering platforms. This builds on previous work exploring multifunctional metasurfaces for polarization conversion and control [29], [30].

## 2 Results

The object of our research is a chiral resonant dielectric metasurface, which consists of Si cylinders in a monoclinic arrangement on a SiO_2_ substrate. Such a metasurface is geometrically chiral, i.e. it does not possess any mirror symmetry: the up-down mirror symmetry is broken by the substrate, and all in-plane mirror symmetries are broken by the monoclinic arrangement itself. Circular dichroism studies of this metasurface can be found in Ref. [28].

Realization of a polarization transformation can be conveniently visualized on the unit Poincaré sphere [5], [31] via the following three parameters:

(1) $$\begin{array}{ll} \tau & =|e_{x}{|}^{2}-|e_{y}{|}^{2}=\frac{I_{\text{V}}-I_{\text{H}}}{I_{\text{V}}+I_{\text{H}}},\\ \chi & =2\;Re\left(e_{x}^{*}e_{y}\right)=\frac{I_{\text{D}}-I_{\text{A}}}{I_{\text{D}}+I_{\text{A}}},\\ \sigma & =2\;Im\left(e_{x}^{*}e_{y}\right)=\frac{I_{\text{L}}-I_{\text{R}}}{I_{\text{L}}+I_{\text{R}}}. \end{array}$$

**Figure 2:**
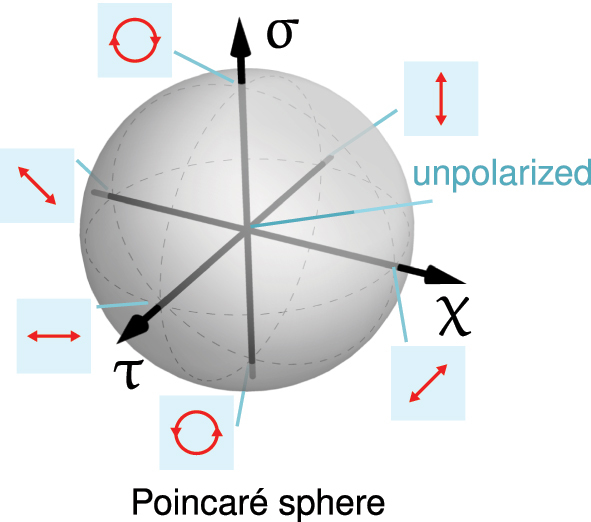
Poincarè sphere and illustration of the polarization states.

These parameters show the degrees of the vertical/horizontal (*τ*), diagonal/anti-diagonal (*χ*), and right-hand/left-hand circular polarizations (*σ*), see Figure 2. Here, the complex components of the electric field *e*_*x*_ and *e*_*y*_ are normalized so that |*e*_*x*_|^2^ + |*e*_*y*_|^2^ = 1. In the far-field plane the field is transverse, i.e. *e*_*z*_ = 0. Experimentally, Stokes parameters are calculated by performing six different intensity measurements: vertical and horizontal *I*_V/H_, diagonal and anti-diagonal *I*_D/A_, polarized on the right and left circular *I*_R/L_. The normalized Stokes parameters satisfy *τ* ^2^ + *χ* ^2^ + *σ* ^2^ ≤ 1, where “ < ” is achieved for the partially or fully unpolarized signal.

**Figure 3:**
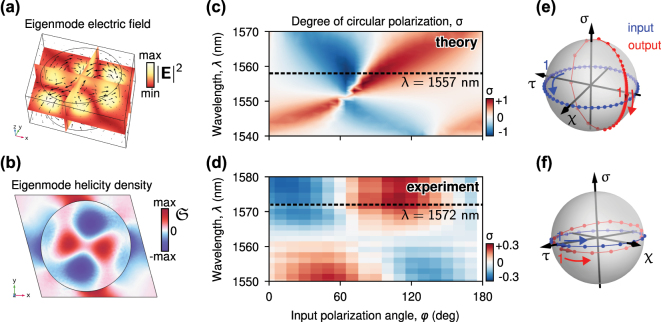
Linear chiral optical properties. Theoretical electric field (a) and helicity density (b) of the excited eigenmode at 1,557 nm. Theoretical (c) and experimental (d) degree of circular polarization *σ* dependence on input polarization angle and wavelength. Output theoretical (e) and experimental (f) polarization state (red dots) for various input linear polarization angles (blue dots) plotted on the Poincare sphere.

The metasurface exhibits resonant behavior. The design parameters are as follows: the meta-atoms are cylinders with a height of 400 nm and a radius of 430 nm, composed of silicon (Si); the monoclinic arrangement is defined by two lattice vectors with lengths *a* = 1,100 nm and *b* = 1,000 nm, and a lattice angle of 105° (Figure 1a); the substrate is made of SiO_2_. We find several eigenmodes of the systems in the vicinity of the wavelength telecommunication range. In particular, there is an eigenmode with a resonant wavelength of 1,553 nm and *Q*-factor of *Q* ≈ 100. We show its electric field distribution in Figure 3a. Geometrically chiral metasurface supports chiral eigenmodes, however, their high level of chirality – selective interaction with right and left circularly polarized output channels – is not guaranteed, and has to be engineered [28], [32], [33], [34]. It was shown in Ref. [28] that the eigenmode in Figure 3a exhibits strong chiral properties, which manifests in a strong circular dichroism.

Additionally to the transmission properties of the resonant chiral metasruface with monoclinic lattice arrangement, we also shed the light on its *local* properties. Helicity density is a local property of the electromagnetic field distribution. In particular it has direct application in chiral sensing [35], [36], which is often referred as one of the promising applications of the chiral metasurfaces. For monochromatic field at frequency *ω* it is written as [37], [38], [39].

(2) $$S=\frac{1}{2\omega c}Im\left(H^{*}\cdot E\right),$$

where **E** and **H** are the electric and magnetic fields, *c* is the speed of light. The quantity (2) characterizes the difference between the numbers of right-hand and left-hand circularly polarized photons. We plot the distribution of helicity density of the eigenmode in Figure 3b.

Next, we study the polarization transformation of such metasurface for the linearly polarized input light in the linear and nonlinear regime, i.e. polarization state of the third harmonic generation signal.

### 2.1 Polarization transformation in linear transmission

Here we examine the manifestation of the chiral mode of choice in the context of polarization transformation for the linearly polarized input. We set the input field to be a monochromatic linearly polarized plane wave at frequency *ω* and wave vector **k** = − $\hat{z}$ *k* (we assume a e^−i*ωt*^ time dependence):

(3) $$E_{\text{in}}^{\left(\omega \right)}=E_{0}\left(\hat{x}\cos\varphi +\hat{y}\sin\varphi \right)e^{-ikz},$$

where angle *φ* shows linear polarization orientation with respect to the *x*-axis, $\hat{x}$ , $\hat{y}$ , $\hat{z}$ are the Cartesian unit vectors. Based on Eq. (1), this implies the input Stokes parameters to be

(4) $$\tau_{\text{in}}=\cos\left(2\varphi \right),\;\chi_{\text{in}}=\sin\left(2\varphi \right),\;\sigma_{\text{in}}=0.$$

We incrementally change polarization angle *φ* in the range [0, *π*] with a constant step Δ*φ*, as the results are *π*-periodic.

Adjusting the polarization angle *φ* and the wavelength *λ* of the incident wave, we examine the polarization of the outgoing signal (Figure 3c). Near the chiral resonance, the circular polarization degree *σ* is strongly dependent on *λ* and *φ*. At certain *λ* and *φ*, the transmitted field approaches circular polarization, |*σ*| ≈ 1. Although the input polarization states (4) are evenly spaced, the output states are not. This is illustrated on the Poincarè sphere in Figure 3e. In particular, the output states form a circle on the sphere, indicated by red dots.

To validate our theoretical prediction regarding the polarization change, we measure all Stokes parameters of light transmitted through the metasurface taking into account Eq. (1) (see Supplemental Material for experimental details). The experimentally determined values of *σ*, as a function of the wavelength and the input polarization angle, are presented in Figure 3d. The output light exhibits elliptical polarization around the optical resonance. The behavior observed qualitatively agrees with the theoretical protection. However, the degree of circular polarization varies from −0.18 to 0.26 at 1,572 nm. The output polarization state is illustrated in Figure 3f by red dots, while the input polarization state is also depicted by blue dots. The experimental pattern is similar to the theoretical prediction. The difference between theoretical and experimental results is primarily attributed to fabrication imperfections and the broad range of incident light wave vectors in the experiment, which result in different mode excitations to the theoretical prediction. We provide theoretical and experimental results for wider range in Supplementary Materials.

### 2.2 Polarization engineering of the third harmonic generation

At higher intensities, the electron oscillations within a dielectric structure become *anharmonic*, which can be effectively described using the extended Lorentz model [40]. In its bulk crystalline form, silicon is a centrosymmetric material, making second harmonic generation (SHG) symmetry-forbidden (except surface effects [41]). Therefore, the third harmonic generation (THG) was examined to demonstrate the nonlinear behavior of the metasurface. The nonlinear polarization current responsible for THG can be expressed as $P^{\left(3\omega \right)}=\varepsilon_{0}{\hat{\chi }}^{\left(3\right)}E^{\left(\omega \right)}E^{\left(\omega \right)}E^{\left(\omega \right)}$ , where *ɛ*_0_ is the dielectric constant, **E** ^(*ω*)^ is the electric field at the fundamental frequency, and ${\hat{\chi }}^{\left(3\right)}$ is the fourth-rank nonlinear susceptibility tensor. Silicon has space group *m*3*m*, which results in only 21 nonzero elements in ${\hat{\chi }}^{\left(3\right)}$ with only 4 independent [40]. While it is possible to find values of each component experimentally in some approximations, their values are usually of the same order of magnitude [42], [43]. For simplicity, we assume all non-zero components to be equal, e.g. as it is done in [44]. Moreover, in Supplementary Materials we show that even approximation of isotropic nonlinear response gives practically the same results. To model THG, we employ the undepleted pump approximation and simulate the process in COMSOL Multiphysics using a two-step approach. In this framework, the nonlinear polarization **P** ^(3*ω*)^ is used as the initial condition for solving the higher harmonic wave equations.

The coupling strength of the incident field to the resonant mode strongly depends on the overlap integral between the two [28], [32], [34], [44]. This coupling shows a strong polarization dependence, providing different field distribution at the fundamental frequency **E** ^(*ω*)^ for different input polarizations, and hence different **P** ^(3*ω*)^ and third harmonic response.

**Figure 4:**
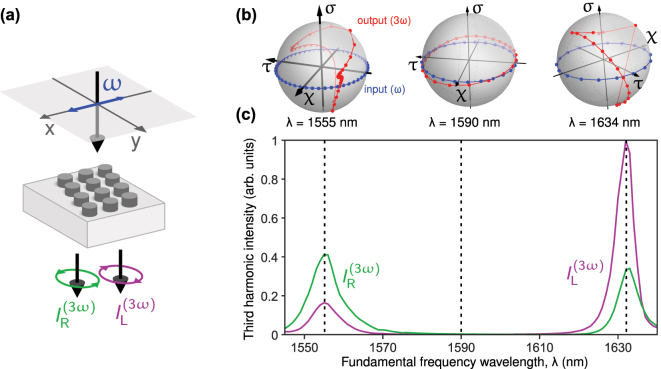
Nonlinear theory. (a) Third harmonic intensity output from a linearly polarized beam along *x*-axis. (b) Poincarè spheres which show simulated polarization states of the third harmonic output taken at different wavelengths for linear polarization input at different angles on the fundamental frequency. (c) The green and magenta lines are the intensity of the right and left circular polarization intensities of the third harmonic signal, respectively.

We evaluate the nonlinear numerical results by calculating the THG emitted along the zeroth order for a linearly polarized pump and decomposing THG transmission signal into the right- and left-circular polarized components, $I_{\text{R}}^{\left(3\omega \right)}$ and $I_{\text{L}}^{\left(3\omega \right)}$ (Figure 4a). The wavelength-dependent THG intensities for right- and left-circular polarized components are shown in Figure 4c by the green and magenta lines, respectively. The results exhibit a pronounced resonant behavior revealing significant enhancement of the THG at the vicinity of the structure resonances. Remarkably, for the first resonance (1,555 nm) the right-circular polarized component dominates, while for the second resonance (1,634 nm) the left-circular polarized component becomes dominant. Next, we investigate the effect of varying the polarization angle of the pump at specific wavelengths and extract all polarization parameters for the THG. The simulated polarization states are represented on the Poincarè spheres in Figure 4b. Near the chiral resonances, the polarization trajectories for pump wavelengths of 1,555 nm and 1,634 nm exhibit high degrees of circular polarization, with |*σ*| values approaching 1. This indicates that highly circularly polarized THG is generated near these resonances. Unlike the linear case, the polarization points do not lie within a single plane and lack a clear pattern. This complexity likely results from the high density of resonant chiral states in the vicinity of 3*ω* (see Supplementary Material), where each mode shows a slightly different coupling coefficient – i.e. an overlap integral between the fundamental harmonic field and the high harmonic resonance – that varies significantly with the linear polarization angle with the resonance at the fundamental harmonic, as the field intensity is less homogeneous. In contrast, for wavelengths away from the resonances (e.g. 1,590 nm, as shown in Figure 4b), the output polarization closely mimics the input polarization, indicating the significance of the chiral resonance at the fundamental harmonic. This aspect requires further investigation and is not fully covered in the current paper.

**Figure 5:**
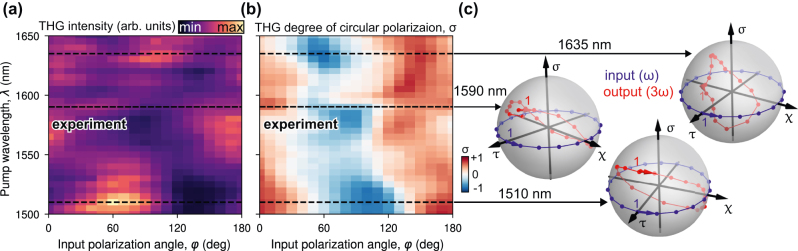
Experimental nonlinear polarization properties. THG intensity (a) and degree of circular polarization (b) from the metasurface as a function of the pump wavelength and polarization angle. (c) Poincarè spheres illustrating polarization states of the output THG at 1,510 nm, 1,590 nm and 1,635 nm wavelengths for linear polarized input, varying linear polarization angle at the fundamental harmonic.

To test the theoretical predictions, we measure the THG from the metasurface (see Supplemental Material for more details). The laser wavelength was tuned from 1,500 to 1,730 nm at 5 nm intervals, while the polarization angle was varied from 0° to 180° with 10°. For each combination of these parameters, we record the THG spectra and extract the maximum values. Figure 5a shows the maximum THG value as a function of the pump wavelength and input polarization angle. We observe THG enhancement in both expected and unexpected spectral regions. The exact reason of such discrepancy between numerical simulations and experimental observation is rather unknown. However we speculate here on possible reasons:

(i) imperfections in fabrications are much more noticeable on the scale of *λ* ^(3*ω*)^/*n* ≃ 125 nm (the typical wavelength of the TH signal inside metasurface material with refractive index *n*), specifically at the cylinders edges where the high-harmonic modes are mostly localized as they resemble whispery gallery modes;
(ii) difference in the refractive index dispersion used in simulations and dispersion of the real sample, as the change of the refractive index tend to shift the position of the resonances;
(iii) deviation of excitation shape from a plane wave, which was used in the theoretical calculations.

Numerical simulations of the THG intensity signal of the range shown in Figure 5a and typical mode profiles at 3*ω* frequencies are shown in Supplementary Materials.

To investigate the degree of circular polarization *σ* we extract this parameter from the THG data taking into account Eq. (1) – the dependence on the pump wavelength and input polarization angle is shown in Figure 5b. The results reveal complex dependencies with significant changes in the polarization state as the pump polarization angle. We provide a possible justification in the Supplementary Material. We further extract the polarization parameters for the resonant wavelengths 1,510 nm and 1,635 nm. These values are plotted on Poincarè spheres, showing *σ* ranges of −0.73 to 0.71 at the wavelength of 1,635 nm and −0.74 to 0.70 at 1,510 nm (Figure 5c). Like the theoretical results, these points do not lie in a single plane as we would expect from the experimental results, and reach higher values for circular polarization than those in the linear regime. In the non-resonant regime at 1,590 nm, while *σ* values are non-zero (−0.39 to 0.58), the low output intensity means other fabrication errors were likely to play a large role. The deviation between the experimental and theoretical results is attributed to the same factors as in the linear case. Additionally, the THG exhibits greater sensitivity to all possible imperfections and pump parameters compared to the linear regime. Considering the impact of the modes at the THG wavelength, it is challenging to fully replicate the simulated result in a real finite sample.

## 3 Conclusions

We have studied the effect of resonances on the polarization conversion in chiral dielectric metasurfaces, for both linear and nonlinear regimes. We have demonstrated that metasurfaces composed of a monoclinic lattice of achiral meta-atoms possess a chiral response that can be employed for active polarization engineering. We have verified that such an intrinsic chirality of the metasurface can transform input linearly polarized light into elliptically polarized light, and we have demonstrated that this effect can be used to control the polarization of the generated third harmonic field. We believe that our results provide the first step in exploring polarization transformations in the nonlinear regime for resonant chiral metasurfaces, and they lay the foundation for future work to optimize such phenomena for applications in chiral sensing, chirality encoding, and chiral imaging.

## Supplementary Material

Supplementary Material Details
