# Supplementary material for: Monoclinic nonlinear metasurfaces for resonant engineering of polarization states
Source: Nanophotonics. 2025 Apr 23;14(23):4145–51. doi: 10.1515/nanoph-2025-0019 (PMC12617705; doi:10.1515/nanoph-2025-0019)
Supplement: Supplementary file 1 — Supplementary Material Details [file j_nanoph-2025-0019_suppl_001.pdf]

Supplementary Material
Monoclinic nonlinear metasurfaces for resonant engineering of polarization states

Ivan Toftul,[1,*] Dhruv Hariharan,[1,†] Pavel Tonkaev,[1,†] Fangxing Lai,[2] Qinghai Song,[2] and Yuri Kivshar[1,‡]

[1] *Nonlinear Physics Center, Research School of Physics, Australian National University, Canberra ACT 2601, Australia*
[2] *Ministry of Industry and Information Technology Key Lab of Micro-Nano Optoelectronic Information System, Guangdong Provincial Key Laboratory of Semiconductor Optoelectronic Materials and Intelligent Photonic Systems, Harbin Institute of Technology, Shenzhen 518055, P. R. China*

S1. NUMERICAL SIMULATIONS

All numerical simulations were performed in the Wave Optics module of COMSOL Multiphysics. The near-field distributions, resonant wavelengths, and Q-factors are simulated using the eigenfrequency solver. Linear and nonlinear transmission simulations are simulated in the frequency domain. The metasurface was placed on a semi-infinite substrate surrounded by a perfectly matched layer mimicking an infinite region in the vertical direction. The simulation area is the unit cell with Floquet periodic boundary conditions which simulates an infinite size of the metasurface in a transverse plane. The dispersion of the refractive index of Si is extracted from the ellipsometry data (see SM of Ref. [1]), while that of SiO_2 is taken from Refs. [2, 3].

The background field is set manually via custom code using Fresnel equations. The third harmonic generation (THG) is calculated in the undepleted pump approximation using the domain polarization feature [4]. The nonlinear polarization current is calculated as $P_i^{(3\omega)} = \varepsilon_0 \hat{\chi}^{(3)}_{ijkm} E_j^{(\omega)} E_k^{(\omega)} E_m^{(\omega)}$, where $\hat{\chi}^{(3)}$ tensor has 21 nonzero elements based on the Si symmetry class $m3m$ (227-th space group) [5, Table 1.5.4]:

$$\begin{aligned}
&xxxx = yyyy = zzzz, \\
&yyzz = zzyy = zzxx = xxzz = xxyy = yyxx, \\
&yzyz = zyzy = zxzx = xzxz = xyxy = yxyx, \\
&yzzy = zyyz = zxxz = xzzx = xyyx = yxxy.
\end{aligned} \tag{S1}$$

Here $ijkm$ is the shorthand for $\hat{\chi}^{(3)}_{ijkm}$ for $i, j, k, m = x, y, z$. Among 21 nonzero elements only 4 are independent. We assume that the crystallographic axes are aligned with the metasurface grating direction and incident field direction, i.e. with the base Cartesian unit vectors $(\hat{\mathbf{x}}, \hat{\mathbf{y}}, \hat{\mathbf{z}})$. While it is possible to find values of each component experimentally

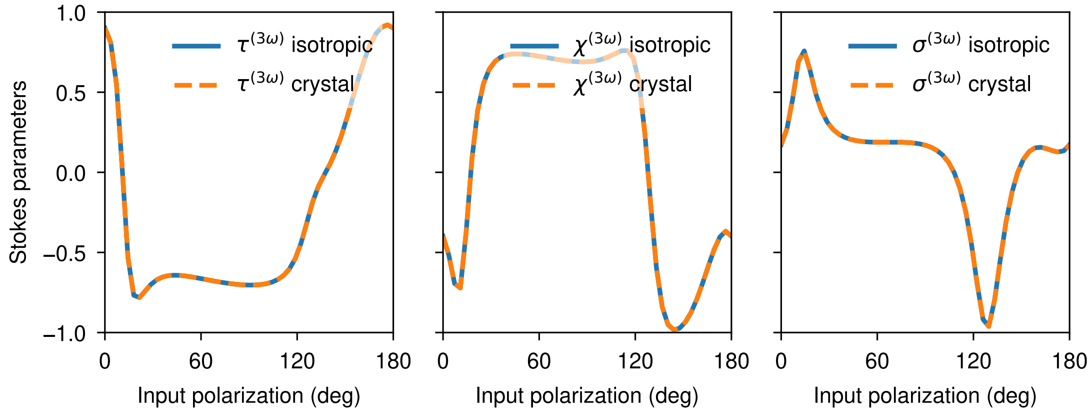

FIG. S1. Stokes parameters of the THG signal for the $\lambda_{\text{pump}} = 1555$ nm for crystalline Si with $\hat{\chi}^{(3)}$ defined by (S1) and for isotropic Si, for which nonlinear polarization is defined by Eq. (S2).

* Equal contribution; ivan.toftul@anu.edu.au
† Equal contribution
‡ yuri.kivshar@anu.edu.au

in some approximations, their values are of the same order of magnitude [6, 7]. For simplicity we set these to be equal. Furthermore, even approximation of the isotropic Si works well in this particular scenario:

$$\mathbf{P}^{(3\omega)} \approx \varepsilon_0 \chi^{(3)} \left(\mathbf{E}^{(\omega)}\right)^2 \mathbf{E}^{(\omega)}, \tag{S2}$$

where $\chi^{(3)}$ is a scalar. On Fig. S1 we compare the polarization output of THG signal for the $\lambda_{\text{pump}} = 1555$ nm. We find that there are no practical difference in the results.

Once the total fields are calculated for the specific background field, $\mathbf{E}_{\text{bg}}$, the complex co-polarized transmission amplitude coefficients are calculated as $t^{(n\omega)}_{\substack{\text{R}\\\text{L}}} = \left\langle \hat{\mathbf{e}}_{\substack{\text{R}\\\text{L}}} \middle| \mathbf{E}^{(n\omega)}_{\text{bg}} \right\rangle = \frac{1}{A} \iint\limits_{A} \hat{\mathbf{e}}^*_{\pm} \cdot \mathbf{E}^{(n\omega)}_{\text{bg}}(x, y, z_0) \mathrm{d}x\mathrm{d}y$, where A is the area of the $z = z_0$ plane located at the edge of the simulation area from the opposite side of excitation, and $\hat{\mathbf{e}}_{\pm} = (\hat{\mathbf{x}} \pm \mathrm{i}\hat{\mathbf{y}})/\sqrt{2}$ are the unit vectors in the circuar polarization basis. Integration over surface A averages the output signal over the angles, so it gives only the 0-th diffraction order. Finally, the transmission coefficients are calculated as $T^{(\omega)}_{\substack{\text{R}\\\text{L}}} = \frac{n_{\text{subs}}}{n_{\text{host}}} \left| t^{(\omega)}_{\substack{\text{R}\\\text{L}}} \right|^2$, and the output harmonic intensity is $I^{(3\omega)}_{\substack{\text{R}\\\text{L}}} \propto \left| t^{(3\omega)}_{\substack{\text{R}\\\text{L}}} \right|^2$, where the proportionality coefficient is unimportant within the scope of this work.

S2. SAMPLE FABRICATION

The silicon metasurface was fabricated with a combined process of electron-beam (E-beam) evaporation, E-beam lithography and inductive coupled plasma (ICP) etching. Basically, 400 nm silicon film was deposited by E-beam evaporation, and then covered with 26 nm Cr layer and 80 nm E-beam resist (PMMA A2). The nanostructures designed in PMMA are patterned via an E-beam aligner (Raith E-line, 30 kV) and developed in MIBK: IPA for 60 s. Taking the PMMA as a mask, the Cr layer was etched with O_2 and Cl_2 (Gas flow of O_2 is 12.5 sccm, gas flow of Cl_2 is 37.5 sccm, pressure is 12 mTorr) in ICP. The Si metasurface was achieved by further etching the Si layer with a mixture of SF_6 and C_4F_8 (Gas flow of SF_6 is 35 sccm, gas flow of C_4F_8 is 40 sccm, pressure is 15 mTorr) in ICP and removing the Cr layer with chromium etchant.

S3. OPTICAL EXPERIMENTS

For the transmission measurements, a quartz tungsten-halogen lamp was used as the light source (Fig. S6**a**). The emitted light passed through a linear polarizer mounted on the motorized rotatable stage enabling precise adjustment of the linear polarization angle. Next, the polarized light is focused onto the sample by a CF_2 lens with a focal length of 50 mm. The transmitted signal after passing through the sample was collected by a 20X Mitutoyo Plan Apo NIR objective lens. Then, the light passed through a quarter-wave plate and a linear polarizer, both mounted on rotatable stages, enabling the extraction of all polarization parameters through various combinations of orientations. Subsequently, the light collected was coupled into an optical fiber by an aspherical lens with a focal length of 8 mm and delivered to the NIRQuest Ocean Optics spectrometer.

For nonlinear transmission measurement, a laser system comprising a femtosecond laser (Ekspla Femtolux 3) operating at a wavelength of 1030 nm and an optical parametric amplifier (MIROPA Hotlight Systems) was used as THG pump source (Fig. S6**b**). The optical parameter amplifier generated tunable NIR radiation in the range of 1500–1700 nm. The laser pulses had a duration of 250 fs, a repetition rate of 5.14 MHz, an average power of 20 mW and spot size of diameter 25 μm. To selectively excite specific wavelengths, the laser was tuned in 10 nm increments across the wavelength range, and the resulting output was measured at each selected wavelength. The NIR laser radiation was linearly polarized with vertical linear polarizer. Next, the polarization was rotated by a half-wave plate mounted on a rotatable stage. The polarized laser beam was focused onto the sample by a CaF_2 lens with a focal length of 50 mm. The transmitted THG signal was collected after passing through the sample by a 20X Mitutoyo Plan Apo NIR objective lens. Subsequently, the THG signal was directed through a Thorlabs superachromatic quarter-wave plate (range 325 – 1100 nm) and a linear polarizer, both mounted on rotatable stages, allowing for the extraction of all polarization parameters by varying their orientations. Finally, the THG signal was coupled into an optical fiber using an aspherical lens with a focal length of 8 nm and delivered to the QE Pro Ocean Optics spectrometer.

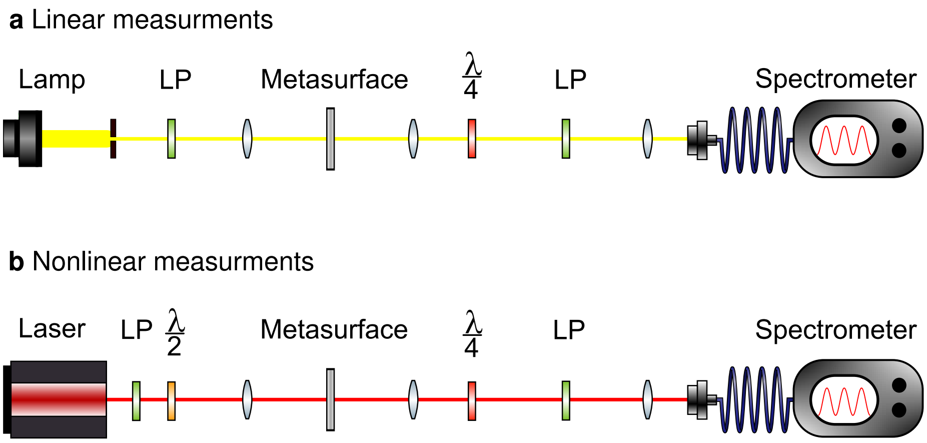

FIG. S2. **Linear and nonlinear experimental setups.** Linear measurements **(a)** were taken with a quartz tungsten-halogen lamp light source. A linear polarizer (LP) was used to convert the unpolarized light to linearly polarized. Nonlinear measurements **(b)** were taken from a femtosecond laser. An LP was used to ensure the laser light was fully linearly polarized before the half-waveplate rotated its polarization. Various lenses were used to align the system, focus the light onto the sample and couple it to the fiber spectrometer.

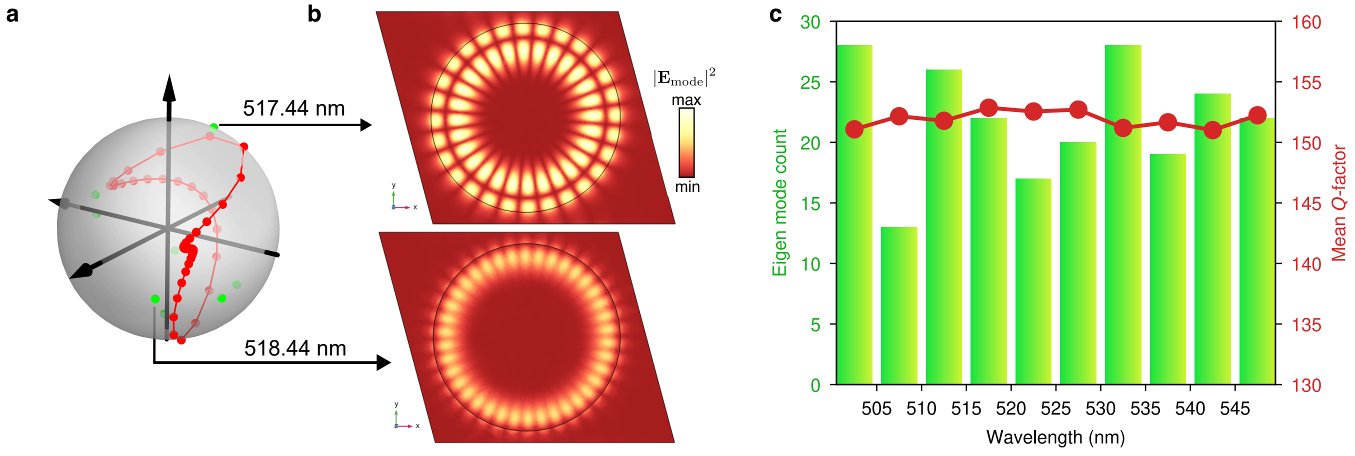

FIG. S3. **a** Poincarè sphere for 1555 nm THG with eigenmodes. The green points correspond to the polarization states of the eigenmodes within 2 nm of the third harmonic frequency. The red line is the theoretical polarization states of the output. **b** Examples of mode profiles corresponding to 517.44 nm and 518.44 nm. **c** Histogram of the eigen mode modes with 5 nm width bin and corresponding mean Q-factor. Simulations were done for the $\varepsilon_{\mathrm{SiO_2}} = 2.14$ and $\varepsilon_{\mathrm{Si}} = 21.5 - \mathrm{i}0.14$.

S4. THIRD-HARMONIC GENERATION MODE

Fig. S3**a** shows the TH output state for the 1555 nm, and shows that the possible excited resonant modes all lie close to this output path. This indicates that the complex shape of the path is likely a result of different coupling amplitudes to different modes at various points on the sphere. For example, the "loop" is positioned in between three different excited modes on the sphere, which likely contribute more than the other modes positioned at other locations. However, the complete analyses of the output THG polarization profile contains a lot of challanges which are outside of the scope of current work. Fig. S3**c** shows the mode density in the relevant to the THG spectral region, which indicates a huge number of mode one has to potentially consider.

Fig. S4 shows a strong dependence of helicity density at the third harmonic for different input polarization angles.

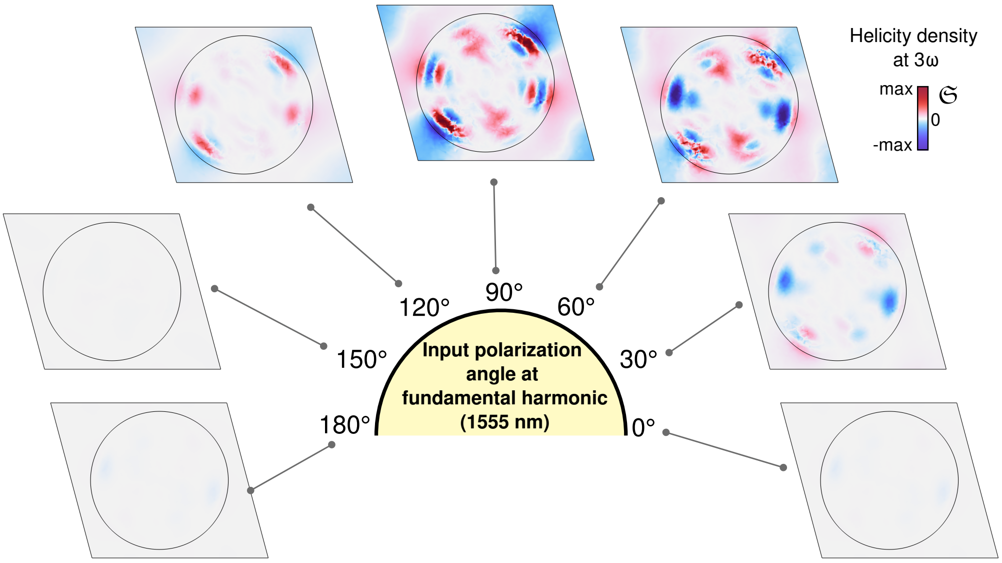

FIG. S4. Helicity density at the third harmonic for different input polarization angles. Min and max values of the color bar are of the same scale for each input polarization angle.

S5. STOKES PARAMETER GRAPHS FOR ALL REPORTED MODES

Figure S6 presents the Stokes parameter graphs for all examined modes, comparing theoretical and experimental results. Panels **(a)** and **(b)** show the Stokes parameters for the linear case, highlighting similarities and differences between theory and experiment. Panels **(c)** and **(d)** display the corresponding results for the nonlinear case, demonstrating the distinct polarization behavior of THG.

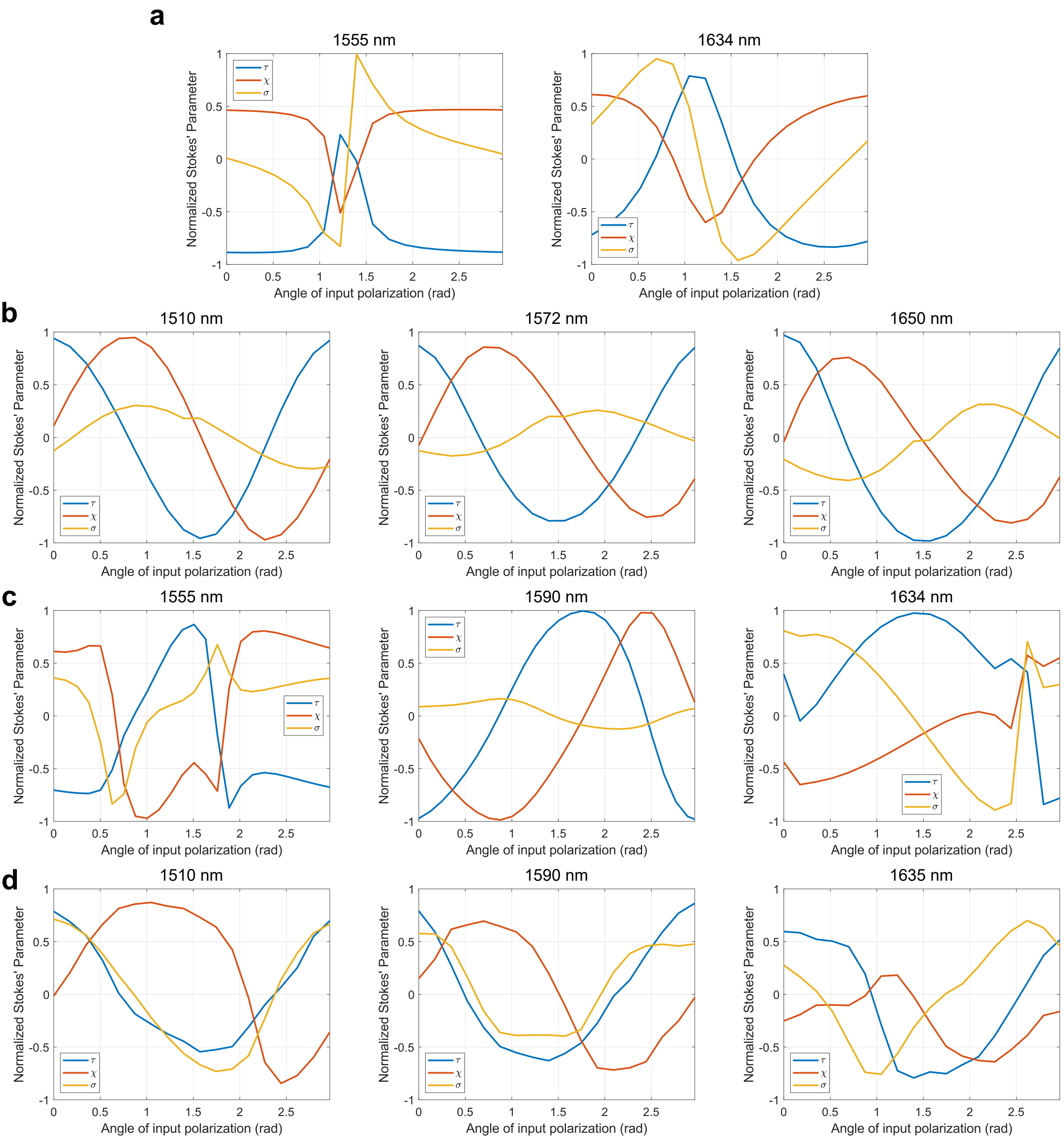

FIG. S5. **Graphs of Stokes parameters for all examined modes.** Linear **(a)** theoretical and **(b)** experimental results. Nonlinear **(c)** theoretical and **(d)** experimental results.

S6. THEORETICAL AND EXPERIMENTAL HEATMAPS

Figure S6 provides a detailed comparison of theoretical and experimental heatmaps for circular polarization and THG efficiency. Panel **(a)** illustrates the calculated THG efficiency across the studied wavelength range, while panel **(b)** presents the theoretical circular polarization distribution. Panels **(c)** and **(d)** focus on the linear mode at 1634 nm, with the theoretical and experimental circular polarization heatmaps showing strong correspondence and emphasizing the role of chiral resonances.

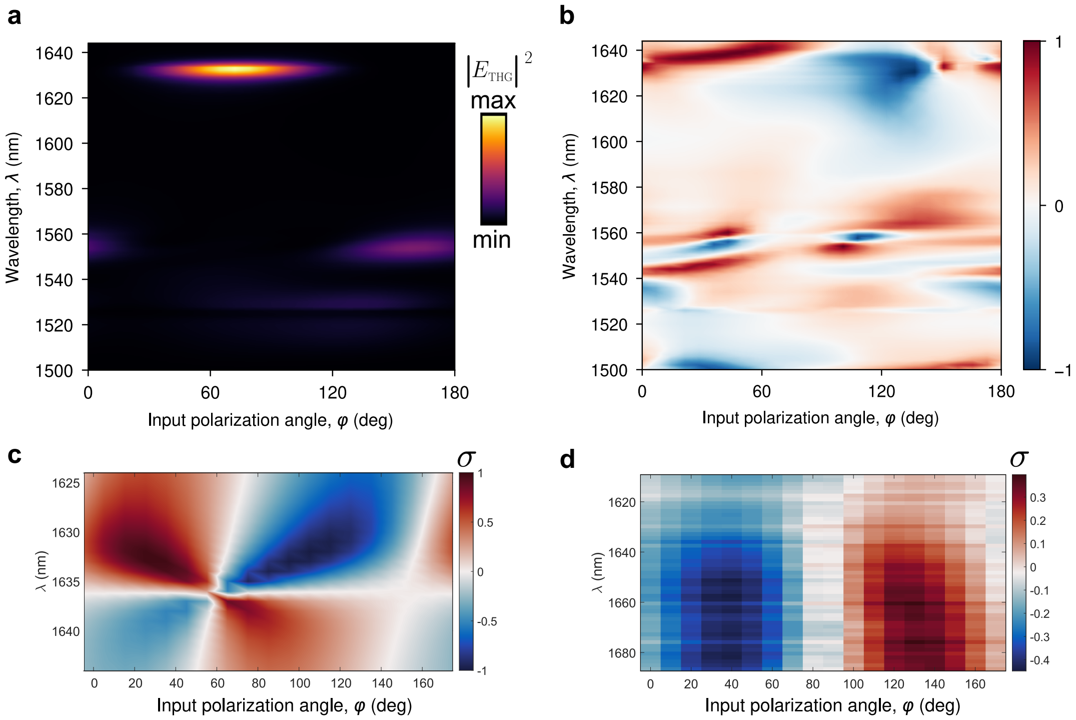

FIG. S6. **Heatmaps for σ and THG.** Theoretical **(a)** THG efficiency and **(b)** circular polarization for wavelength range studied . Theoretical **(c)** and experimental **(d)** circular polarization heatmaps for the linear mode at 1634 nm.

[1] I. Toftul, P. Tonkaev, K. Koshelev, F. Lai, Q. Song, M. Gorkunov, and Y. Kivshar, Chiral Dichroism in Resonant Metasurfaces with Monoclinic Lattices, Phys. Rev. Lett. **133**, 216901 (2024).

[2] I. H. Malitson, Interspecimen comparison of the refractive index of fused silica, JOSA **55**, 1205 (1965).

[3] M. N. Polyanskiy, Refractiveindex.info database of optical constants, Sci. Data **11**, 1 (2024).

[4] Second Harmonic Generation in the Frequency Domain (2024), [Online; accessed 22. Apr. 2024].

[5] R. W. Boyd, A. L. Gaeta, and E. Giese, *Springer Handbook of Atomic, Molecular, and Optical Physics* (Springer, 2008) pp. 1097–1110.

[6] Y.-H. Zhang, H. Liu, Z.-G. Chen, G. Jia, and C. Ren, Research on third-order susceptibility tensor of silicon at telecom wavelength, in *Proceedings Volume 7656, 5th International Symposium on Advanced Optical Manufacturing and Testing Technologies: Optical Test and Measurement Technology and Equipment*, Vol. 7656 (SPIE, 2010) pp. 1673–1677.

[7] D. J. Moss, H. M. van Driel, and J. E. Sipe, Dispersion in the anisotropy of optical third-harmonic generation in silicon, Opt. Lett. **14**, 57 (1989).
